# Effect of chitosan edible coating with *Laurus nobilis* extract on shelf life of cashew

**DOI:** 10.1002/fsn3.630

**Published:** 2018-03-23

**Authors:** Behnaz Azimzadeh, Mahshid Jahadi

**Affiliations:** ^1^ Department of Food Science and Technology Faculty of Agriculture, Isfahan (Khorasgan) Branch Islamic Azad University Isfahan Iran

**Keywords:** cashew, chitosan, edible coating, *Laurus nobilis*, shelf life

## Abstract

This study tested the effects of the application of *Laurus nobilis* aqueous extract and edible coating of chitosan had on the chemical, microbial, and sensory attributes of cashew's shelf life. An aqueous extract of *L. nobilis* leaf (0, 0.5, and 1% *w/w*) was added to chitosan solution (0, 0.5, 1% *w/w*) in the cashew's coating. Cashews were dipped into the coating solution and were kept in polyethylene terephthalate containers. The result showed that chitosan and aqueous extract of *L. nobilis* coating had significant effects on peroxide and thiobarbituric acid value (*p* < .05). There was significant reduction in the growth of mold/yeast and mesophilic bacteria with higher concentration of chitosan and *L. nobilis* aqueous extract (*p* < .05). The results of this study show chitosan aqueous extract of *L. nobilis* coating could be an effective preservative method for extending shelf life and improving the stability of cashew.

## 1 INTRODUCTION

During the past decade, the production of cashews has increased significantly as cashews gained global notoriety as a valuable nutritional source. Cashew kernels alone have considerable nutritional value, including protein (21%), fat (47%), moisture (5.9%), carbohydrate (22%), phosphorus, calcium, iron, and other mineral elements. The most important vitamins in cashews are vitamins E, D, and A, which help assimilate fats and increase the immune system (Yahaya, Taiwo, & Shittu, 2012). Cashew nuts are rich in polyunsaturated fatty acid, containing **ω**_9_ (59%–61%) and **ω**_6_ (17%–21%) (Akinhanmi & Atasie, 2008; Soares, Vasconcelos, & Camelo, 2012). Also, they are a rich source of essential fatty acids, which also makes them prone to oxidation. One common method for improving the stability of lipid‐containing foods such as cashews is the use of edible coating (Chlebowska, Gniewoz, & Gazseweska, 2008; Ghasemzadeh, Karbassi, & Ghoddousi, 2008; Haq, Alam, & Hasnian, 2013).

Edible coating is when edible substances are poured directly on the food surface in thin layers (Kang, Kim, & You, 2013). The edible coating is easily consumable; meanwhile, it avoids food quality deterioration by acting as a barrier against moisture, oxygen transferring, and dissolving substances. Thus, the edible coating increases the shelf life of fat‐rich foods and eliminates deterioration reactions (Maghsoudlou, Maghsoudlou, Khomeiri, & Ghorbani, 2012). They can be a suitable barrier to oxygenation. Also, preventing oxygen penetration slows down the lipid oxidation process in nuts containing unsaturated fatty acids. Chitosan (B‐(1,4)‐2 amino‐2‐deoxy‐d‐glucopyranose) is one of the most popular edible coatings in the food industry. It is a polysaccharide extracted from the shells of crustaceans, such as shrimp, crab, and other sea crustaceans (Gavhane Yogeshkumar, Gurav Atul, & Yadav Adhikarao, 2013). Chitosan is the second most abundant, nontoxic polysaccharide in nature after cellulose. Chitosan shows antifungal and antibacterial properties, which are believed to be originated from its polycationic nature. Generally, the edible coating in nuts could be used as a vehicle for additives such as antioxidants (Haq et al., 2013). The addition of a powerful antioxidant to the coating can work in synergy. There is recognition that natural antioxidants are better than synthetic for maintaining health (Kang et al., 2013).

It has been confirmed that the *Laurus nobles* aqueous extract has the high numbers of phenolic compounds with strong antioxidant activities (Cherrat, Espina, Bakkali, Garica‐gonzalo, & Pagan, 2013; Hinneburg, Dorman, & Hiltunen, 2006; Jamshidi, Hashemi, & Ebrahimzadeh, 2007). Lauraceae composed of 32 genera and about 2000–2500 species. Laurel, a member of the family, is an evergreen tree or shrub, which is grown in many warm parts of the world like the Southern Meditation region, Europe, and the United States. Especially, it has medicinal properties and is used to treat the symptoms of gastrointestinal problems, such as epigastric bloating, impaired digestion, eructation, and flatulence, and to treat epilepsy (Jamshidi et al., 2007). The objective of this study was to develop to chitosan‐based edible coating and to evaluate the efficiency of natural antioxidant (*Laurus nobilis*) addition in preventing lipid oxidation in the cashew nut kernel.

## 2 MATERIALS AND METHODS

### 2.1 Reagents

Shelled cashews were purchased at a local supermarket. Chitosan (medium weight) was purchased from Sigma‐Aldrich Company. Glycerol, a plasticizer of edible coating solutions, was purchased from Jabarfar Company located in Isfahan—Iran. Chloroform, glacial acetic acid, petroleum ether, potassium iodide, and trichloroacetic acid were purchased from Merck (Germany).

### 2.2 Preparation of aqueous extract of *Laurus nobilis*

Leaves of *L. nobilis* were collected by authors from an agricultural research center in Isfahan, Iran. After cleaning and washing the leaves, they were dried in a dark place and grounded to obtain the aqueous extract. Five grams of ground materials was soaked in 100 ml of double‐distilled water for 24 hr at room temperature on the shaker. Next day, they were centrifuged at 2500 g for 4 min and then segregated by filter paper (Whatman No. 1). The solvent was evaporated under reduced pressure at 45°C in rotary vacuum evaporator for 40 min. The extracts were kept in dark bottles at 4°C (Hinneburg et al., 2006).

### 2.3 Coating solution preparation

The solution consisting of chitosan powder (0, 0.5, and 1 mg in 100 ml of water), glycerol (0.25% of the usage of chitosan powder), acetic acid (1/100 ml of water), and *L. nobilis* aqueous (0, 0.5, 1% into 100 ml) was added to sterile double‐distilled water and mixed by a magnetic stirrer at 65°C for 3 hr. The pH of the final solution was set at 5.5 by the addition of sodium bicarbonate (Maghsoudlou et al., 2012)

### 2.4 Cashew coating procedure

For each treatment, 80 g of cashew nuts was placed in a mesh container and immersed in chitosan solutions for 30–40 s. Coated nuts were dried in the oven at 45°C for 20 hr.

### 2.5 Storage conditions and sampling

After coating and drying, cashew nut samples were packaged in 150 × 135 × 30 mm polyethylene terephthalate (PET) containers. The samples were stored at 37 ± 1°C during 90 days for measuring of chemical characteristic (peroxide and thiobarbituric acid value and sensible) and at 27 ± 3°C (the room temperature at 60th day) for microbial characteristic.

### 2.6 Chemical methods

#### 2.6.1 Peroxide value (POV)

The oil was extracted by soaking the cashew grounds (50 g) with 100 ml of petroleum ether in light proof room for 11 hr on the flat shaker. Then, the solvent was centrifuged (4000 g for 15 min). The fine cashew powder and pieces were completely separated using a filter paper (Whatman No. 1). The petroleum ether in the separated supernatant was removed using evaporation equipment (IKA, German) at 45°C. 30 ml of chloroform/acetic acid (2:3) (v/v) was added into the 5 g of extracted lipid specimen. Then, the 1.5 ml of saturated potassium iodide solution was added to the chloroform/acetic acid/lipid solution. The solution was stored in the dark room for 1 min. Then, 30 ml of distilled water was added to the solution and was shaken strongly. 1 ml of starch solution (1 g/100 ml of water) was added as an indicator, and the mixture was titrated with 0.005 N. POV was calculated according to the following formula.

$$\mathrm{Peroxide}\;\mathrm{value}=\left(\frac{\mathrm{miliequivalent}\;\mathrm{peroxide}}{\mathrm{kg}\;\mathrm{sample}}\right)=\frac{(S-B)\times N\times 1000}{\mathrm{WS}}$$

Herein, B was the volume (ml) of titrant for blank, *S* was the volume (ml) of titrant for the sample, *N* was the normality (moles equivalent/L) of the solution, and *WS* was the weight (*g*) of the sample (Kang et al., 2013).

#### 2.6.2 Thiobarbituric acid value

Thiobarbituric acid (TBARS) test is a method commonly used as a measurement of the products of the second lipolysis, especially monoaldehydes. Smashed cashew (1 g) was mixed in 20 ml of distilled water and 20 ml of TBA (0.5 g/100 ml of water) containing trichloroacetic acid (20 g/100 ml of water). After stirring, the mixture's color changed to yellow in the water bath at 85°C for 1.5 hr. The reaction was stopped by putting the mixture into the ice box for 10 min. The sample was centrifuged at 4000 rpm for 4 min, and the supernatant was removed and filtered (Whatman No. 1). The optical density of separated supernatant was read at 532 nm with a spectrophotometer (Unico from Korea) (Kang et al., 2013).

### 2.7 Microbial analysis

A sample from each treatment (5 g) was blended in the sterile blender and homogenized in 45 ml of sterile distilled water. After shaking (1 min), aliquots of 0.5 ml were inoculated in replicating plate of different media. Plate count agar (PCA) and yeast glucose chloramphenicol agar (YGC) were inoculated for total mesophilic aerobic bacterial and fungal (yeast and mold) analysis, respectively. The PCA and YGC plate were incubated at 37°C and 25°C for 24 hr, respectively (Oranusi & Braid, 2012).

### 2.8 Sensory analysis

Sensory evaluation was performed with seven hedonic scales. In this experiment, 30 panelists (15 female and 15 male) were selected and each panelist tested all nine treatments. Five factors, such as color and bitterness, hardness, flavor acceptability, and general acceptability of the product, were evaluated by the panelists.

### 2.9 Statistical analysis

The study was replicated three times. Data were analyzed using SPSS version 17. Analysis of variance (ANOVA) followed by Duncan's multiple range tests was used to distinguish the treatment at *p* < .05.

## 3 RESULTS AND DISCUSSION

### 3.1 Effect of edible coating on lipid oxidation of cashew

The effect of the coating formulations on the POV in cashews is shown in Table 1. POV increased significantly during storage in all the samples from initial stage of storage (first day) to the end of storage (90 days; *p* < .05). However, this increase was significantly lower in coated samples compared to control. The effectiveness of the *L. nobilis* coating is highly dependent on the concentration of the extract. Also, with the increasing content of the chitosan and *L. nobilis* extract in the coating solution, the POV decreased significantly (*p* < .05). Among the coated groups, it was observed that those without chitosan and/or *L. nobilis* had higher POV than those with chitosan and/or *L. nobilis* during 90 days of storage.

**Table 1:** Peroxide value (m Eq kg^−1^ cashew) changes of cashew coated with various coating formulations during storage

Treatment		Time (day)					
*Laurus nobilis* extract (%)	Chitosan (%)	0	15	30	45	60	90
0	0	0.12 ± 0.04 Abc	0.21 ± 0.03 Ab	0.22 ± 0.03 Ab	0.27 ± 0.04 Ab	0.34 ± 0 Aa	0.39 ± 0.01 Aa
0	0.5	0.06 ± 0.05 Abb	0.22 ± 0.11 Aa	0.14 ± 0 Bab	0.17 ± 0.04 Bab	0.17 ± 0.04 Bcab	0.14 ± 0.07 Cdab
0	1	0.13 ± 0.04 Abc	0.09 ± 0 Ac	0.14 ± 0 Bbc	0.22 ± 0.04 ABa	0.22 ± 0.04 Ba	0.19 ± 0 BCDab
0.5	0	0.04 ± 0.01 Bb	0.07 ± 0.04 Ab	0.16 ± 0.02 Bab	0.22 ± 0.03 ABa	0.22 ± 0.1 Ba	0.2 ± 0.01 Bca
0.5	0.5	0.03 ± 0 Bb	0.04 ± 0 Ab	0.04 ± 0 Cb	0.04 ± 0 Cb	0.07 ± 0.04 Cb	0.12 ± 0.04 Da
0.5	1	0.11 ± 0.02 Aba	0.2 ± 0.27 Aa	0.09 ± 0 BCa	0.2 ± 0.01 ABa	0.23 ± 0.02 Ba	0.22 ± 0.04 Bca
1	0	0.07 ± 0.04 Abb	0.22 ± 0.04 Aa	0.24 ± 0/07 Aa	0.24 ± 0.07 ABa	0.27 ± 0.04 Aba	0.27 ± 0.04 Ba
1	0.5	0.12 ± 0.11 Abab	0.19 ± 0 Aa	0.04 ± 0 Cb	0.17 ± 0.04 Ba	0.2 ± 0.01 Ba	0.19 ± 0 BCDa
1	1	0.17 ± 0.03 Aa	0.12 ± 0.02 Aab	0.07 ± 0.03 Cb	0.16 ± 0.05 Ba	0.17 ± 0.04 Bca	0.14 ± 0 Cdab

^(a–z)^ Within a column, different letters indicate significant differences (*p *<* *.05); ^(A–Z)^ Within a row, different letters indicate significant differences (*p *<* *.05).

The TBARS value is another method that is widely used as an index of rancidity. The presence of TBARS is due to second stage of auto‐oxidation and represents compounds that are responsible for off flavor or odor produced during storage of greasy food (Kang et al., 2013). Change in TBARS values over the storage time is indicated in Table 2. The TBARS values consistently increased, during storage time in all of the experimental groups. For all storage times, the maximum TBARS value occurred in the uncoated control group in comparison with other samples, where the minimum was observed in the sample that included 1% chitosan and *L. nobilis* extract. TBARS values of the cashews coated with *L. nobilis* extract were significantly lower than those groups without *L. nobilis* extract. In the case of cashews coated with *L. nobilis* and/or chitosan, TBRSA values were lower than non‐*L. nobilis* and/or chitosan samples during 90 days during storage. In these two experiments, POV and TBARS values, which indicated the level of lipid oxidation, increased steadily over storage time. The numerical values of POV and TBARS in the coated groups were significantly lower than those of the uncoated control group. This indicates that the edible coating delayed lipid oxidation of cashews by protecting them from oxygen exposure during storage (Bourtoom, 2008). Other factors could account for the antioxidant properties of *L. nobilis* extract. Antioxidant components within the coated matrix structure interact by creating cross‐linking leading to the improved structure and reduction in the delivery of oxygen inside the cover (Haq et al., 2013).

**Table 2:** TBARS (Absorbance: 532) changes of walnut kernels coated with various coating formulations during storage

Treatment		Time (day)					
*Laurus nobilis* extract (%)	Chitosan (%)	0	15	30	45	60	90
0	0	0.44 ± 0.026 Aba	0.139 ± 0.014Cc	0.079 ± 0.004 CDd	0.172 ± 0.023 Bc	0.142 ± 0.016Ac	0.254 ± 0.045Ab
0	0.5	0.163 ± 0.006Bcab	0.056 ± 0.006Dc	0.119 ± 0.045 BCb	0.045 ± 0.004 EFc	0.058 ± 0 Bc	0.168 ± 0.002ABa
0	1	0.508 ± 0.161Ab	0.740 ± 0.033Aa	0.129 ± 0.006 BCc	0.281 ± 0.017 Ac	0.156 ± 0.078Ac	0.230 ± 0.042ABc
0.5	0	0.675 ± 0.196Aa	0.352 ± 0.016Bb	0.195 ± 0.003 Abc	0.137 ± 0.024 Cc	0.027 ± 0.004 Bc	0.222 ± 0.028ABbc
0.5	0.5	0.491 ± 0.268Aba	0.148 ± 0.001 Cb	0.147 ± 0.059 ABb	0.077 ± 0.003 Db	0.035 ± 0.004Bb	0.222 ± 0.01ABab
0.5	1	0.547 ± 0.17Aa	0.024 ± 0.001Db	0.05 ± 0.006 Db	0.034 ± 0.002Fb	0.047 ± 0.001Bb	0.203 ± 0.057ABb
1	0	0.427 ± 0.037Aba	0.315 ± 0.029Bb	0.032 ± 0.001Dd	0.032 ± 0.004 Fd	0.0028 ± 0.009Bd	0.148 ± 0.068Bc
1	0.5	0.598 ± 0.18Aa	0.154 ± 0.028Cb	0.165 ± 0.004 ABb	0.066 ± 0.005 Dec	0.005 ± 0Bc	0.137 ± 0.054Bb
1	1	0.103 ± 0.004Cb	0.041 ± 0.008 Dc	0.17 ± 0.018ABa	0.045 ± 0.008 EFc	0.046 ± 0.001Bc	0.180 ± 0.025ABa

^(a–z)^ Within a column, different letters indicate significant differences (*p *<* *.05); ^(A–Z)^ Within a row, different letters indicate significant differences (*p *<* *.05).

### 3.2 Effect of edible coating on microbial quality of cashew

Microbiological quality of samples on the bases of total mesophilic aerobic bacteria and fungi (log 10 cfu/g) is shown in Table 3. Count of total mesophilic aerobic bacteria and fungi on uncoated and coated cashew at the production time (0 day) demonstrated that the coated (coating solution without any chitosan and *L. nobilis*) sample had lower counts. The effect of acetic acid in the coating solution in lowering the count was significant due to its antimicrobial effect, the amount of bacteria, and mold growth during the storage in control samples are still significantly increased (*p* < .05; Table 3). In the sample containing the edible chitosan coating, the mold and bacteria growth were lower than control sample and this effect is heightened when the chitosan is conjunction with *L. nobilis*. There was a significant difference in antimicrobial effects of various concentrations of chitosan. Based on the result of this study, cashew with 1% chitosan and 1% *L. nobilis* was the best formula for decreasing the microbial and fungi growth. This result shows that with increasing the content of chitosan and *L. nobilis* in coating formulation, the fungi development and bacteria development were decreased significantly (*p* < .05).

**Table 3:** Mesophilic aerobic bacteria and fungi populations in coated and uncoated cashew during the storage

Treatment			Total count (log 10 cfu/g)	
*Laurus nobilis* extract (%)	Chitosan (%)	Time (day)	Bacteria	Yeast and mold
Uncoated cashew		0	7.30 ± 0.04^a^	7.49 ± 0.02^a^
0	0	0	6.50 ± 0.14^bc^	6.52 ± 0.11^b^
0	0	60	6.86 ± 0.07^ab^	5.33 ± 0.57^c^
0	0.5	60	6.29 ± 0.26^bcd^	4.20 ± 0.14^de^
0.5	0	60	6.11 ± 0.07^cdef^	4.77 ± 0.32 ^cd^
0.5	0.5	60	5.93 ± 0.67^cdef^	4.12 ± 0.16^de^
0.5	1	60	5.55 ± 0.13^ef^	4.70 ± 0.36 ^cd^
1	0	60	5.85 ± 0.12^cdef^	4.07 ± 0.58^de^
1	0.5	60	5.77 ± 0.02^def^	3.44 ± 0.06^ef^
1	1	60	5.50 ± 0.49^f^	3.20 ± 0.29^f^

Means within columns with different letters are significantly different (*p* < .05).

Chitosan is a nontoxic and antimicrobial compound, which has dual functional effects on the product's shelf life. It also has direct effect on fungi growth and different function of the immunity such as chitosan retention, which reduces the fungal cell wall synthesis of protein inhibitors (Bourtoom, 2008; Gavhane Yogeshkumar et al., 2013; Hernandez‐Munoz, Almenar, & Valle, 2008). One of the reasons for the antimicrobial characteristic of chitosan is its positively charged amino group, which interacts with the negatively charged microbial cell membranes, leading to the leakage of proteinaceous and other intracellular constituents of microorganisms (Pranoto, Rakshit, & Salokhel, 2005). Chitosan has high antimicrobial properties in a wide range of pathogenic microorganisms and spoilage bacteria, such as gram‐positive and gram‐negative bacteria and fungi (Gavhane Yogeshkumar et al., 2013; Jiang & Li, 2001). These coatings are more effective in the presence of antimicrobial additives such as *L. nobilis* extract, which has high antioxidant and antibacterial effects (Keskin, Oskay, & Oskay, 2010; Rakshit & Ramalingam, 2010).

### 3.3 Effect of edible coating on sensory evaluation of cashew

The sensory evaluation test was described for cashews by 30 panelists (male and female) who were selected for this purpose. The selection criteria were based on the ability to detect and describe five basic characteristics (color, hardness, bitterness, flavor, and general acceptability). The result of sensory evaluation in Table 4 indicates that all treatments had changed during the storage in comparison with the first day.

**Table 4:** Sensory evaluation samples of cashews covered by chitosan and *Laurus nobilis* extract stored at 27 ± 3°C on the sixtieth day

Treatment		Evaluated characteristic				
*L. nobilis* extract (%)	Chitosan (%)	Color	Hardness	Bitterness	Flavor	General acceptability
0	0	1.77 ± 0.43^a^	3.77 ± 1.45^a^	4.87 ± 0.43^a^	4.97 ± 1^a^	5.13 ± 0.82^a^
0	0.5	1.7 ± 0.43^ab^	3.87 ± 1.223^a^	4.60 ± 0.56^abc^	4.67 ± 0.92^abc^	4.77 ± 0.97^ab^
0	1	1.33 ± 0.48 ^cd^	3.25 ± 1.97^a^	4.33 ± 0.88 ^cd^	4.30 ± 1.21^bc^	4.30 ± 1.24^b^
0.5	0	1.47 ± 0.51^bcd^	3.80 ± 1.19^a^	4.73 ± 0.58^ab^	4.73 ± 0.83^ab^	4.83 ± 0.83^ab^
0.5	0.5	1.50 ± 0.51^bcd^	4.10 ± 1.45^a^	4.67 ± 0.66^abc^	4.67 ± 1.03^abc^	5 ± 0.98^a^
0.5	1	1.27 ± 0.45^d^	4.43 ± 0.94^a^	4.20 ± 0.92^d^	4.47 ± 0.97^abc^	4.57 ± 1.14^ab^
1	0	1.60 ± 0.50^abc^	4.23 ± 1.36^a^	4.83 ± 0.38^a^	4.80 ± 0.85^ab^	4.83 ± 0.91^ab^
1	0.5	1.47 ± 0.51^bcd^	3.77 ± 1.25^a^	4.70 ± 0.6^abc^	4.93 ± 1.11^a^	5 ± 0.95^a^
1	1	1.23 ± 0.41^d^	4.10 ± 1.03^a^	4.40 ± 0^bcd^	4.13 ± 1.20^c^	4.30 ± 1.24^b^

Means within columns with different letters are significantly different (*p *<* *.05).

Based on Table 4, the color of samples was strongly increased when the concentration of *L. nobilis* extract in edible coating increased. In other words, the color of the samples which were coated by 1% *L. nobilis* extract was darker than lower concentration and increasing the chitosan did not have any significant effect on the color of samples. The color analysis of the sample describes cream (1) and light cream (2). In this research, by increasing the chitosan concentration, the color of coated cashew did not change. The result of the present research corresponds with the result of Maghsoudlou et al. (2012). Chitosan coating, by blocking the oxidation reaction, prevents color change (Bourtoom, 2008). By increasing the *L. nobilis* concentration to 0.5% in coating formulations, we did not have any significant effect on color, but increasing the extract to 1% caused the color of cashew increased and became cream because increasing the extract concentration in edible coating made the cashew darker in color. The texture of cashew is significantly affected by moisture content (Maghsoudlou et al., 2012).

In addition, the hardness of cashew did not increase significantly by adding the chitosan and *L. nobilis* concentration (high hardness (6), medium hardness (5), low hardness (4), low softness (3), medium softness (2), and high softness (1)). The content of chitosan did not have any significant effect on bitterness of coated cashew. Meanwhile, by increasing the *L. nobilis* until 0.5%, the bitterness was not increased significantly, but 1% *L. nobilis* created bitterness feeling in the panelist (without any bitterness (5), little bitterness (4), medium bitterness (3), high bitterness (2), very high bitterness (1)). However, by increasing the *L. nobilis* concentration to 1%, the flavor acceptability decreased significantly. The coated samples had low ratings in terms of flavor acceptability in comparison with other samples. This result shows the increasing of *L. nobilis* concentration has negative correlation with flavor (highly tasty (6), medium tasty (5), not too tasty (4), highly insipid (3), medium insipid (2), not too insipid (1). General acceptability was another parameter that was measured. The increasing content of chitosan to 1% and *L. nobilis* to 0.5% did not have significant effects on general acceptability, but increasing *L. nobilis* to 1% on the edible coating resulted in decreased general acceptability (Table 4). As a result, the best treatments were selected by panelist have 0.5% extraction of *L. nobilis* and 0.5%–1% chitosan. Result of sensory evaluation indicated that all treatment had changed during the storage in comparison with the first day. Oxygen is one of the most important factors that can affect the quality of fatty food. Diffusion of oxygen to the cashew's tissue resulted in browning reactions and undesirable color changes (Maghsoudlou et al., 2012). When cashews absorb moisture from the surrounding environment, they become a soft and sticky texture that is not desirable for consumers. During the storage of cashews, chitosan coating acts as a barrier against moisture to penetrate into the tissue's sample as well as keeping moisture constant within the tissue (Bourtoom, 2008; Song & Cheng, 2014). As a result, the hardness was maintained during storage. In this study, we observed that by increasing the chitosan, the bitterness in cashew was not increased, but by increasing the *L. nobilis* extract to 1%, the bitterness was increased, because of high concentration of the extract. Also, by increasing the concentration of chitosan to 1% and *L. nobilis* to 0.5%, there was not any significant difference in flavor samples of cashew. To sum up, it can be concluded that increasing the chitosan concentration to 1% did not have any significant difference on general acceptability between coated samples. Additionally, adding the *L. nobilis* extract until 0.5% did not make any change on general acceptability, but 1% *L. nobilis* resulted in an undesirable taste and desirable general accessibility between samples.

## 4 CONCLUSIONS

The results of this work indicate that aqueous extract of *L. nobilis* leaf and chitosan can be used as natural edible coating to increase the shelf life improve the stability of cashew. This natural edible coating effectively delayed lipid oxidation in comparison with the uncoated cashew. Also, the chitosan coating with *L. nobilis* extract placed on the surface of the product can reduce microbial load and demonstrated antimicrobial effects on mesophilic bacteria and fungi. This coverage does not have any impact on color, hardness, bitterness, flavor, and general accessibility.

## CONFLICT OF INTEREST

The authors declare that they do not have any conflict of interest.
